# An evolutionary game-theoretic analysis of construction workers' unsafe behavior: Considering incentive and risk loss

**DOI:** 10.3389/fpubh.2022.991994

**Published:** 2022-09-13

**Authors:** Jianling Huang, Yidan Wu, Yang Han, Yang Yin, Guangbo Gao, Huihua Chen

**Affiliations:** Department of Engineering Management, School of Civil Engineering, Central South University, Changsha, China

**Keywords:** workers' unsafe behavior, evolutionary game theory, system dynamics, incentive, risk loss

## Abstract

The behavior of construction workers has a significant impact on the overall safety climate of a project. The purpose of this paper is to figure out the evolutionary pattern of workers' unsafe behavior and to minimize its occurrence. We constructed a two-sided evolutionary game model consisting of workers and managers to explore the focal point of interest, strategy equilibrium conditions, and behavior evolution process. The experimental results of stability analysis and system dynamics show that there are two stable states in all four cases, (Safe behavior, Negative management) as well as (Unsafe behavior, Negative management). The lower the initial willingness of workers to behave unsafely, the faster they reach a safe steady state. By contrast, managers' strategy choices have a certain lag. Workers are discouraged from choosing unsafe behavior under both the positive incentive of raising bonuses and the negative incentive of raising fines. And the sensitivity of the two incentives is similar. For indirect effect risk loss, when it is effectively controlled during safe construction, workers quickly gravitate toward safe behavior. These findings provide a reference for construction safety management. Several practical suggestions were proposed from three perspectives: the worker, the manager, and the site safety climate, focus on the theme of reducing unsafe behavior and achieving a virtuous cycle of safety climate.

## Introduction

Construction is one of the most dangerous industries in the world because of the harsh working environment, the complexity of the work and the high labor intensity (1). In the U.S., construction-related safety incidents account for 20% of the total, while in the UK and Ireland, they are 25% and 50% (2). The issue of building safety has been a great challenge for academia as well as industry (3). Many countries have made a lot of efforts in construction safety related laws and regulations, management system and other aspects, but the current state of safety management in the construction industry is still unsatisfactory. The annual construction safety accidents and the resulting casualties have been at a high level (4). In China, the number of production and safety accidents in housing and construction projects was 773 in 2019, an increase of 5.3% year-on-year. The biggest safety hazard is “fall from height,” accounting for 53.69% of the total. The remaining three major safety accidents are object strikes, earth collapses and mechanical injuries (5). Construction is a labor-intensive industry with many parties involved, and thus the factors for safety accidents are multifaceted. The exposed and changing environment, insufficient safety awareness and professional quality of workers, improper organization and management, and lack of availability of materials and equipment may all lead to problems in a certain aspect of safety production, and even cause serious consequences such as personnel casualties (6, 7). Among them, numerous studies worldwide have found that the direct cause of safety accidents is various irregular operations and dangerous behaviors of human beings (8–10). Therefore, an in-depth study of construction workers' unsafe behavior (CWUB) is a prerequisite and foundation for solving the safety problems in construction effectively.

Unlike others, the construction industry has unique billing methods, organizational structures and operating conditions (11). Most construction workers (such as steelworkers, carpenters, and tilers) are paid based on the amount of work performed. This feature drives workers to do as much work as possible in order to earn more income, easily leading to violations. For example, the pursuit of higher moving speeds without wearing protective measures or crossing safety distances. Furthermore, workers are as temporary as the construction project, while lacking firm convictions (12). Guided by weak safety awareness and general education, managers' systems and codes have limited restraint on workers. The era of Industry 4.0 has arrived in contemporary society, and intelligence is becoming more and more popular. The existing urban structure and construction methods are also undergoing drastic changes (13). The opposite of the frequent construction accidents is the booming construction market (14). For construction enterprises to survive and grow in the fierce competition, they must focus on the control of production safety accidents and maximize their economic benefits. In the context of high-quality development, safety management is changing from rough to intensive (15). This has prompted companies to upgrade to urban service providers and deepen industrial restructuring. In consideration of the overall safety climate, managers need to improve the safety responsibility system and supervision mode in the process. Given the role of incentive mechanism in facilitating group decision making (16), it is beneficial in disciplining and correcting worker behavior when localized by managers.

Affected by the limited attention, incomplete information collection and processing ability, and uncertainty of the environment, the subjects involved in the CWUB system are limitedly rational (17, 18). Thus, it can be seen that the influence factors of CWUB are multi-faceted (6). In terms of individual emotions, workers who harbor negative psychological states such as fear and anxiety are more likely to engage in unsafe behaviors than those who feel happy and warm (19). Head-mounted equipment is often used to monitor workers' heart rate, energy expenditure and other physiological states, to provide timely feedback and reduce CWUB caused by poor physiology (20, 21). In addition, the resulting psychological problems are often mentioned (22). This confirms that research conducted based on cognitive neurological categories is a key direction for behavioral psychology (23, 24). Motivated intentions tend to guide the development of behavior, and stable emotions are conducive to securing safe behavior. Good cognitive skills help workers learn from safety experiences to identify hazards early (25). Furthermore, Organizational safety climate (26), cognitive biases (27), influence of workmates (28), operating environment (29), resource device readiness (8) and family factors (30) are also reasons why workers' behavior may fluctuate toward violations. Among them, family is considered a source of moral support for workers. Safety hazards are present throughout the life cycle of a construction product (31). Workers' mastery of equipment and technology is a major source of risk and in turn affects their level of hazard perception. The system is not only an important part of the atmosphere, but also controls the spread of group perception (32). Supervision is an important part of the system's implementation. Safety leadership not only directly leads to changes in worker behavior, but also indirectly grows the safety culture (3). Safety education and training help to enhance the self-protection ability of workers, and the site organization and management guarantee a reasonable production order (33). This paper will focus on the mutual effects of managers and workers.

The mechanism of CWUB formation is the interaction of different factors such as organization, individual and group, as well as the path of unsafe behavior under the combined effect of different factors (34). The stress and intensity of a worker is often considered to be a facilitator of CWUB. In contrast, safety climate (35), safety perceptions (36), knowledge and risk tolerance (37) have a positive impact on safe behavior. Management commitment and managerial quality (38) play an important moderating role in workers' behavior between these two types of factors. Under the improper management mode, the reaction chain of CWUB presents “Command failure in construction site–Operation unsafe positions–Accident risk” (39). CWUB has network transmission characteristics (40), and technical operators are at the core of the network. The transmission process is characterized by a distinct subgroup structure within the shift. The proportion of workers who choose unsafe behavior can be viewed as the probability that the group will act unsafely in the current situation.

In reality, due to information asymmetry and market dynamics, the main parties of CWUB will not be in an ideal state of decision making. The evolutionary game theory, in which the actors do not have to be perfectly rational (41), fits into the study of the change process of behavioral decisions under multiple factors (42). In the construction field, the research hotspots focus on the behavioral research of stakeholders such as workers, construction units, government, and procurement units, safety management, corporate capacity enhancement, and sustainable development. Taking advantage of green building, Chen used an evolutionary game approach to make suggestions for builders to improve their competitiveness (43). Wu et al. (44) used an evolutionary game model to study the safety behavior strategies of general contractors and workers. Their study highlighted the importance of safety education and safety climate. Guo et al. (45) introduced the government to form a three-way evolutionary game model based on workers and construction units. The results demonstrated the effectiveness of government incentives and penalties in construction safety management. In CWUB, evolutionary game theory can be used to analyze the effect of factors and reveal the path of behavioral change of the two direct participants. In turn, it explains how equilibrium can be achieved to reduce the occurrence of risky behaviors.

Understanding the formation of CWUB is to reach the prevention and control of behaviors, which is the focus of safety management and the goal of research (46). The critical time, critical area and critical node (47) where the behavior occurs reflect the action pattern of the workers. Up to now, the development of smart construction technology has brought project management to a new stage (48). In terms of safety inspection, BIM, computer vision (49) and image recognition technologies (50) have become an important part of smart sites. Artificial intelligence technology optimizes the function and process of safety inspection to facilitate workers' self-inspection before operation.

Inspired by the above studies, this paper aims to analyse the strategic choices of workers and managers in different situations and improve the safety climate. The existing studies are still inadequate in terms of boundary conditions, threshold definition and transformation process between the two behaviors. We intend to contribute to this area by solving two important questions: effect of initial strategy on CWUB system equilibrium and the effect of external variables on subjects' strategies. Therefore, we used evolutionary game approach and system dynamics model to describe the strategy changes of both subjects under dynamic incentives and risk loss. The evolution results are discussed to provide rational references and practical suggestions to reduce safety accidents and achieve sustainable development.

## Methodology

**Figure 1:**
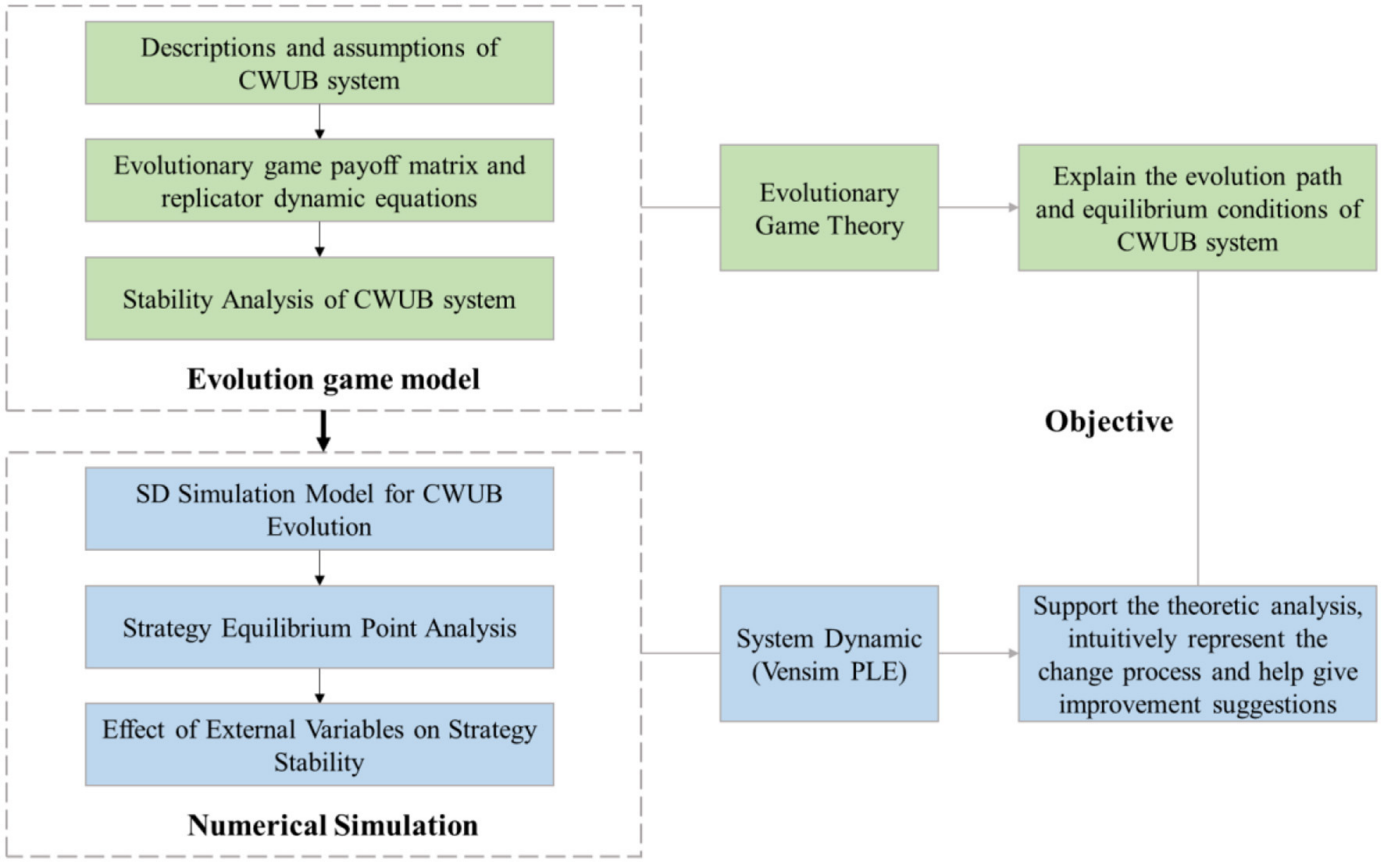
The framework of research methodology.

This paper adopted a hybrid research method including two main steps, as shown in Figure 1. The first step is evolutionary game analysis. The CWUB evolutionary game system is established by calculating the payment matrix and replicator dynamic equations of both sides. Then stability analysis is performed to list the conditions for system stability. The second step is numerical simulation. The evolutionary game model analysis and numerical simulation. System dynamics methods and Vensim PLE are used to explore the effect of initial strategies and external variables on system equilibrium and strategy stability.

### Assumptions

Evolutionary game (51) is about the interaction and iterative process of behavioral strategies. In the game, players choose different behavior strategies, and therefore obtain corresponding “return.” In the safety management system, construction workers and construction managers are close stakeholders, and there is a game problem between the two in terms of strategy choices. While construction managers refer to the front-line one who visit the site or remotely monitor the construction safety and manage the workers. Under the premise of bounded rationality and asymmetric information, it may be difficult for these two stakeholders to make best choices to maximize their own interests. In order to ensure organized construction and avoid unnecessary losses, the manager can choose positive or negative management. Considering the inspection of managers and self-interest, the worker can choose safe or unsafe behavior. These two bodies will adjust their decision-making by predicting the strategic behavior of another, to obtain the final equilibrium point of the game.

According to the actual safety management of construction projects, this paper analyses the game between safety managers and construction workers. Both will try different strategies over time, and settles on a particular stabilization strategy. The strategies of workers groups include “unsafe behavior” and “safe behavior.” Safety management departments have “positive management” and “negative management” strategies.

**Assumption 1**. The main players of the game are workers and managers, and they are both rational economic persons who make decisions based on cost-benefit analysis. In this asymmetrical game process, through constant learning and trial and error, the two heterogeneous groups can settle on their own stabilization strategy. Workers do not consciously always abide by safety operating rules. Their “unsafe behavior” refers to the violation of safety system and safety norms which may cause construction safety accidents.

**Assumption 2**. Suppose the probability that workers adopt unsafe behavior is *α* (*α* ∈ [0, 1]), and the probability that workers adopt safe behavior is (1 − *α*) (52). The normal salary of workers is *R*_1_. When workers do not work according to specifications, the risk loss for workers is $L_{1}^{1}$, while the risk loss for managers is $L_{1}^{2}$. Simultaneously, workers are only penalized *F* if managers use a positive regulatory strategy. When workers comply with specifications, *C*_1_ refers to their safe construction cost, $L_{2}^{1}$ refers to their risk loss, while $L_{2}^{2}$ refers to the risk loss of managers (53). After workers complete the construction safely and efficiently, they will receive subsidies *S* regardless of the strength of management supervision.

**Assumption 3**. Suppose the probability that managers adopt positive management is *β* (*β* ∈ [0, 1]), and the probability that workers adopt negative management is (1 − *β*). The normal salary of managers is *R*_2_. When managers choose to supervise positively, *C*_2_ refers to their safe management cost. When managers choose to supervise negatively, *P* refers to the economic loss caused by negative image and reduction of credibility after the exposure of managerial misconduct.

Theoretically, costs and revenues are also related to several other factors. However, this paper focuses on the effects of incentive mechanisms and the degree of risk on worker behavior. The safety allowance S and the unsafe penalty F represent the strength of the two incentives for managers in the positive and negative directions, respectively. In addition, this study assesses the extent to which management should subsidize the construction side. To simplify and refine the model, we assume that the base wages of workers and managers remain constant and that the risk loss under unsafe behavior is greater than that under safe behavior. The variables and their meanings are shown in Table 1.

**Table 1:** Variables symbol descriptions.

**Variables**	**Descriptions**
*R*_1_	Normal salary of construction workers
*C*_1_	Cost of safe construction
$L_{1}^{1}$	Risk loss of workers under unsafe construction
$L_{2}^{1}$	Risk loss of workers under safe construction
*S*	Safety construction subsidies from the manager to the worker
*F*	Unsafe construction penalties from the worker to the manager
*R*_2_	Normal salary of managers
*C*_2_	Cost of safe management
$L_{1}^{2}$	Risk loss of managers under unsafe construction
$L_{2}^{2}$	Risk loss of managers under safe construction
*P*	Economic loss caused by negative management

### Payoff matrix and replicator dynamic equation

According to the theory of bilateral evolutionary game (54) and the above relationship between workers and managers, this paper lists the payoff matrix of both sides of the game under four different strategy combinations, as shown in Table 2. The payoff of the worker (the “column” player) is represented by the entries preceding the semicolon; that of the manager (the “row” player) is represented by the entries after the semicolon.

**Table 2:** Payoff matrix of both sides of the game.

		**Manager**	
		**Positive management (β)**	**Negative management (1-β)**
Worker	Unsafe behavior (α)	$R_{1}-F-L_{1}^{1}$; $R_{2}-C_{2}+F-L_{1}^{2}$	$R_{1}-L_{1}^{1}$; $R_{2}-L_{1}^{2}-P\;$
	Safe behavior (1-α)	$R_{1}-C_{1}+S-L_{2}^{1}$; $R_{2}-C_{2}-S-L_{2}^{2}$	$R_{1}-C_{1}+S-L_{2}^{1}$; $R_{2}-S-L_{2}^{2}-P\;$

Let *E*_*w*1_ and *E*_*w*2_ represent, respectively the expected earnings of “Unsafe behavior” and “Safe behavior” for workers. According to Table 2, the payoffs of the workers with the two different behavior strategies are as Equations 1 and 2.


(1) $$E_{w1}\;=\beta \left(R_{1}-F-L_{1}^{1}\right)+\left(1-\beta \right)\left(R_{1}-L_{1}^{1}\right)\;$$

(2) $$E_{w2}\;=\beta \left(R_{1}-C_{1}+S-L_{2}^{1}\right)+\left(1-\beta \right)(R_{1}-C_{1}+S-L_{2}^{1})$$

The average earning of the workers is denoted as $\bar{E_{w}}$ showed as Equation 3.


(3) $$\bar{E_{w}}\;=\alpha E_{w1}+\left(1-\alpha \right)E_{w2}$$

Similarly, let *E*_*m*1_ and *E*_*m*2_ represent, respectively the expected earnings of “Positive management” and “Negative management” for managers. According to Table 2, the payoffs of the workers with the two different behavior strategies are as Equations 4 and 5.


(4) $$E_{m1}\;=\;\alpha \left(R_{2}-C_{2}+F-L_{1}^{2}\right)+\left(1-\alpha \right)(R_{2}-C_{2}-S-L_{2}^{2})$$

(5) $$E_{m2}\;=\;\alpha \left(R_{2}-L_{1}^{2}-P\right)+\left(1-\alpha \right)(R_{2}-S-L_{2}^{2}-P\;)$$

The average earning of the managers is denoted as $\bar{E_{m}}$ showed as Equation 6.


(6) $$\bar{E_{m}}\;=\;\beta E_{m1}+\left(1-\beta \right)E_{m2}$$

In unsafety behavior evolution, the two game players will adjust their strategies by learning and a process of trial and error, thus recreating the dynamic replication process described by evolutionary game theory. The replicator dynamics equation is a dynamic differential equation which essentially determines how often a particular strategy is adopted or accepted within a population. Therefore, the replicator dynamic equation of “Unsafe behavior” chosen by workers *F*(*α*) and the replicator dynamic equation of “Positive management” chosen by managers *F*(*β*) are as Equations 7 and 8.


(7) $$\begin{array}{l} F\left(\alpha \right)=\frac{\partial \alpha }{\partial t}=\alpha \left(E_{w1}-E_{w}\right)=\;\alpha \left(1\;-\;\alpha \right)\left(E_{w1}-E_{w2}\right)\\ =\;\alpha \left(1\;-\;\alpha \right)\left[C_{1}+L_{2}^{1}-\;\beta F-S-L_{1}^{1}\right] \end{array}$$

(8) $$\begin{array}{l} F\left(\beta \right)=\frac{\partial \beta }{\partial t}=\beta \left(E_{m1}-E_{m}\right)=\;\beta \left(1\;-\;\beta \right)\left(E_{m1}-E_{m2}\right)\\ =\;\beta \left(1\;-\;\beta \right)[\alpha F+P-C_{2}] \end{array}$$

### Stability analysis

Stability analysis is commonly used to determine the long-run equilibrium strategy of each participant (55). In this paper, dynamic differential analysis is used to obtain the equilibrium strategy of the system. Let the replicator dynamic system be Equation (7) =0 and Equation (8) =0, we can get the equilibrium points E1 = (0, 0), E2 = (1, 0), E3 = (0, 1), E4 = (1, 1). When *P*< *C*_2_ and $S+L_{1}^{1}<C_{1}+L_{2}^{1}$ are hold, we can obtain *F*(*α*_0_) = 0 and *F*(*β*_0_) = 0, at the same time 0 < *α*_0_ < 1 and 0 < *β*_0_ <1, so E5 = (*α*_0_, *β*_0_) is also an equilibrium point, and $\alpha_{0}=\frac{-P+C_{2}}{F}$, $\beta_{0}=\frac{C_{1}+L_{2}^{1}-S-L_{1}^{1}}{F}$. Any initial point and its evolution range must be in a two-dimensional space {(*α*, *β*)|*α* ≤ 1, *β* ≤ 1}. The area surrounded by E1–E5 is the equilibrium solution of the evolutionary game model. However, according to the Jacobian matrix theory, the equilibrium points obtained by the replicator dynamic syste99m are not necessarily the ESS (evolutionary stable strategy) (56, 57). We calculate the determinant det(*J*) and trace *tr*(*J*) of Jacobian matrix to judge the type of the above five equilibrium points. If det(*J*) > 0 and *tr*(*J*) < 0 the equilibrium point is ESS. If det(*J*) > 0 and *tr*(*J*) > 0, the equilibrium point is unstable. Otherwise, the equilibrium point is a saddle point. The Jacobian in this paper is shown in Equation 9.


(9) $$J=\left[\begin{array}{c} \frac{\partial F\left(\alpha \right)}{\partial \alpha }\frac{\partial F\left(\alpha \right)}{\partial \beta }\\ \frac{\partial F\left(\beta \right)}{\partial \alpha }\frac{\partial F\left(\beta \right)}{\partial \beta } \end{array}\right]$$

(10) $$=\left[\begin{array}{c} \left(1\;-2\;\alpha \right)\left[C_{1}+L_{2}^{1}-\beta F-S-L_{1}^{1}\right]\;-\alpha \left(1\;-\;\alpha \right)F\\ \beta \left(1\;-\;\beta \right)F\;\left(1\;-\;2\beta \right)[\alpha F+P-C_{2}] \end{array}\right]$$

Substituting the five equilibrium points of this paper into Equation (9), we obtain their det(*J*)and *tr*(*J*), as shown in Table 3.

**Table 3:** The det(J) and tr(J) of equilibrium points.

**Equilibrium points**	**det(J)**	**tr(J)**
E_1_ (0, 0)	$\left(C_{1}+L_{2}^{1}-S-L_{1}^{1})\;(P-C_{2}\right)$	$C_{1}+L_{2}^{1}+P-S-L_{1}^{1}-C_{2}$
E_2_ (1, 0)	$-\left(C_{1}+L_{2}^{1}-S-L_{1}^{1})\;(F+P-C_{2}\right)$	${-C}_{1}-L_{2}^{1}+S+L_{1}^{1}+F+P$ −*C*_2_
E_3_ (0, 1)	$-\left(C_{1}+L_{2}^{1}-F-S-L_{1}^{1}\right)\;\left(P-C_{2}\right)$	$C_{1}+L_{2}^{1}-F-S-L_{1}^{1}-\;P$ +*C*_2_
E_4_ (1, 1)	$\left(C_{1}+L_{2}^{1}-F-S-L_{1}^{1}\right)$ (*F*+*P*−*C*_2_)	$-C_{1}-L_{2}^{1}+S+L_{1}^{1}-P$ +*C*_2_
E_5_ (*α*_0_, *β*_0_)	$\alpha_{0}\beta_{0}\left(1+\alpha_{0}\beta_{0}-\alpha_{0}-\beta_{0}\right)F^{2}$	0

There is no selection dilemma for symmetric games in which the participants behave identically, and the evolutionary direction of the two sides is inevitable (58). In asymmetric games, the participants have different attributes and characteristics, and the evolution has more possible outcomes (59). In the asymmetric 2 × 2 game of cooperation or defection, the equilibrium points of situations, such as the Chicken (Snowdrift) game and the Stag Hunt game, depend on relative magnitudes of the payoff matrix elements (60). The same is true for the CWUB gaming system with the introduction of incentives. But there are too many factors involved, it is impossible to discuss each case in detail. According to the calculations in Table 3 and the actual construction, the equilibrium points of the model can be discussed in four cases. Because the *tr*(*J*) of E5 is 0, CWUB is unlikely to reach equilibrium at this point. And in the following cases, it is not analyzed.

**Figure 2:**
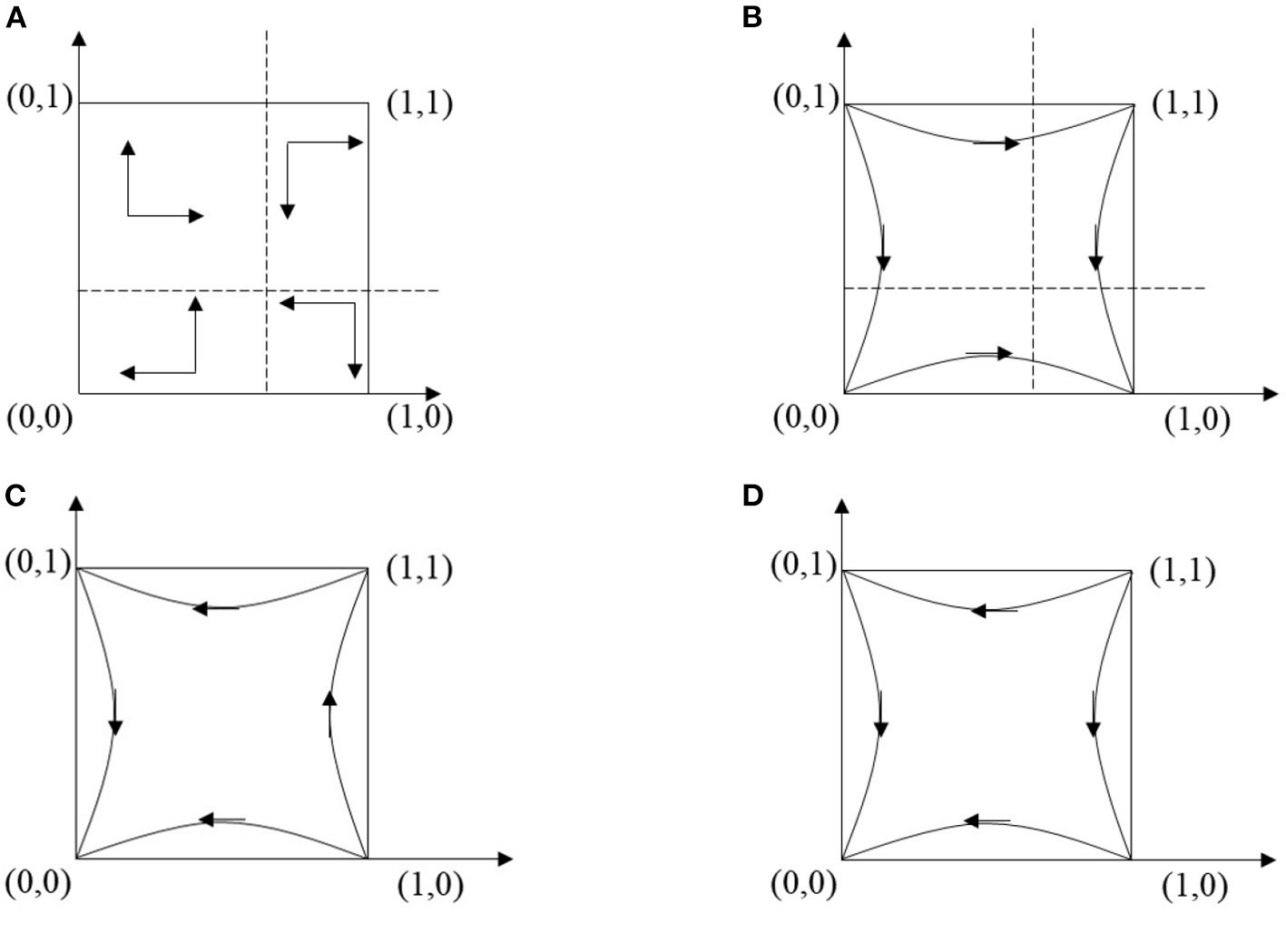
The dynamic evolution process in different cases. **(A)** The dynamic evolution process in case 1. **(B)** The dynamic evolution process in case 2. **(C)** The dynamic evolution process in case 3. **(D)** The dynamic evolution process in case 4.

**Case 1:** $L_{1}^{1}+S<C_{1}+L_{2}^{1}$ and *P*<*C*_2_ < *F* + *P*. The stability analyses of this case are presented in Table 4. The dynamic trend is depicted in Figure 2A. Under this condition, the benefits and expenses of the game subjects are unbalanced. Workers' behavior and managers' attitude show a circular state, oscillating between two options. There is no equilibrium point in the game behavior of both parties, only four saddle points exist. So the evolutionary direction of CWUB is also uncertain.

**Table 4:** Stability analyses of equilibrium points in case 1–4.

**Case**	**Equilibrium points**	**det(*J*)**	**tr(*J*)**	**Stability**
Case 1	E_1_ (0, 0)	-	^*^	Saddle
	E_2_ (1, 0)	-	^*^	Saddle
	E_3_ (0, 1)	-	^*^	Saddle
	E_4_ (1, 1)	-	-	Saddle
Case 2	E_1_ (0, 0)	-	^*^	Saddle
	E_2_ (1, 0)	+	-	ESS
	E_3_ (0, 1)	+	+	Unstable
	E_4_ (1, 1)	-	^*^	Saddle
Case 3	E_1_ (0, 0)	+	-	ESS
	E_2_ (1, 0)	+	+	Unstable
	E_3_ (0, 1)	-	^*^	Saddle
	E_4_ (1, 1)	-	^*^	Saddle
Case 4	E_1_ (0, 0)	+	-	ESS
	E_2_ (1, 0)	-	^*^	Saddle
	E_3_ (0, 1)	-	^*^	Saddle
	E_4_ (1, 1)	+	+	Unstable

“-” indicates a negative calculation result; “+” indicates a positive result; “*” indicates an uncertain result.

**Case 2:** $L_{1}^{1}+S<C_{1}+L_{2}^{1}$ and *P*+*F* < *C*_2_. The dynamic trend is characterized in Figure 2B. When the cost of safe construction is too large and the impact of fines is slight, the behavior of the game subjects will be converging to (unsafe behavior, negative management). There is one stable point, two saddle points and one unstable point in the evolutionary game system, showing a tendency to evolve from unstable point to stable point. In order to pursue greater benefits, workers will choose dangerous behaviors and managers will supervise passively.

**Case 3:** $C_{1}+L_{2}^{1}<L_{1}^{1}+S$ and *P* < *C*_2_ < *F* + *P*. The stability analyses of these equilibrium points, in this situation, are demonstrated in Table 4. The duplicated dynamic trend is represented in Figure 2C. In this case, workers tend to behave safely; meanwhile, managers tend to weak management. When they can't afford excessive fines, groups of workers will prefer safe behavior to stop the damage and earn bonuses. The game will gradually converge to the equilibrium point (0, 0).

**Case 4:** $C_{1}+L_{2}^{1}<L_{1}^{1}+S$ and *P*+*F* < *C*_2_. Table 4 represents the local stability analyses of these equilibrium points in case 4 and Figure 2D displays the duplicated dynamic trend. E2 (1, 0) and E3 (0, 1) are saddle points, E4 (1, 1) is unstable point, and E1 (0, 0) is the ESS. With lower fines and higher bonuses, workers will be inclined to build safely, while reducing the pressure on the regulator to choose a weak management strategy. In a good site atmosphere, the pressure of managers is relieved and the intensity is reduced.

The ideal construction process is one in which workers can consciously engage in safe behaviors, reduce supervisory pressure, and achieve project quality standards while saving labor and material resources. From the above analysis, it can be concluded that under Case 3 and Case 4, construction workers and managers will make the choice of safe behavior and negative management respectively, and the game system will tend to the equilibrium point (0, 0). The skills of workers are mainly acquired by means of teacher-apprentice transmission and self-learning by imitation, which have a large fluctuation and randomness. Without constraints, workers may prefer convenient behavior (potentially dangerous) over compliance with safety regulations. In order for workers to develop consistent safety habits, the manager should control bonuses and penalties. In addition, measures such as the introduction of intelligent management systems (61) can have a large impact on risk loss or other factors, which can affect the outcome of the game between the two parties.

## Numerical simulations

### Construction of the system dynamics model

**Figure 3:**
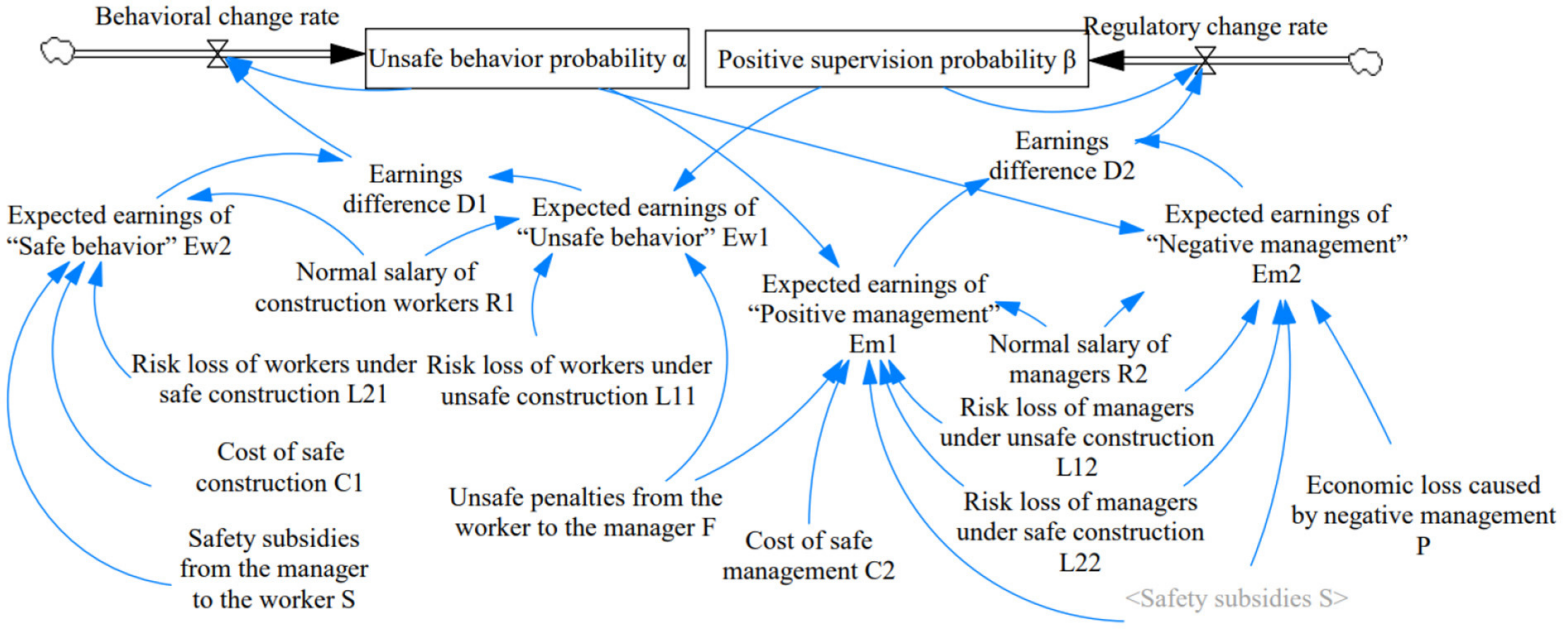
SD Simulation model for CWUB evolution.

In order to analyse more intuitively the influence of different factors on the behavior of construction workers and supervisors, this section conducts repeated game simulations through Vensim PLE. The previously mathematical Equations 1–8 reveal the relationships between the level variables, rate variables, auxiliary variables and exogenous variables, and the system dynamics (SD) model is shown in Figure 3. The 2 rate variables represent the speed of change in the subjects' choice, and their cumulative amount determines the 2 level variables related to “unsafe behavior” and “active management.” Six auxiliary variables include their earnings and earning differentials under diverse strategy choices, and 11 exogenous variables have been shown in Table 1.

Equations 7 and 8 constitute the replicated dynamic system in the evolutionary game model of this paper, which not only determines the direction of the subject's strategy but also explains the relationship between the auxiliary variables and the exogenous variables. The core idea of this simulation approach is to evolve smooth strategies with imitator dynamics that do not necessarily fit perfectly, but can effectively describe the changing patterns of things. The values of the data are not necessarily real, but the trends are well-documented (62). Therefore, for system dynamic models, it is more important to focus on the soundness of the structural design, which can help the safety behavior system to correctly derive behavior trends and driving methods.

The values of the external variables in the SD model are taken mainly considering their sensitivity to construction workers and managers. In the compilation of data and information, they are aggregated and averaged, and the corresponding variable values are obtained by referring to relevant literature studies (63, 64). According to Equations 7 and 8, not all factors play a significant role in the result. Therefore, to improve the efficiency of the study, this paper conducts an assignment analysis for the key influencing factors related to the replication dynamic equations. According to theories related to risk management, workers will incur less losses when using safe behaviors, so $L_{1}^{1}>L_{2}^{1}$. When the builder constructs safely, the cost includes the use of personal protective equipment (PPE), safety warning items, heavy machinery detection, safe power consumption and new products such as smart site and green technology. Generally, it will be higher than the manager's supervision cost. The variables need to satisfy the theoretical analysis, but their scaling is not necessarily constant. This brings out differences in the modeling results as well. So, to improve fault tolerance, two sets of decisive variable values used in the numerical simulations are shown in Table 5.

**Table 5:** The variable values.

**Variables**	***C*** **_1_**	** $L_{1}^{1}$ **	** $L_{2}^{1}$ **	***S***	***F***	***C*** **_2_**	***P***
Set 1	1.5	0.5	0.3	1.2	1	1.3	0.8
Set 2	1.5	0.8	0.4	1.2	1	1.3	0.8

### Strategy equilibrium point analysis

**Figure 4:**
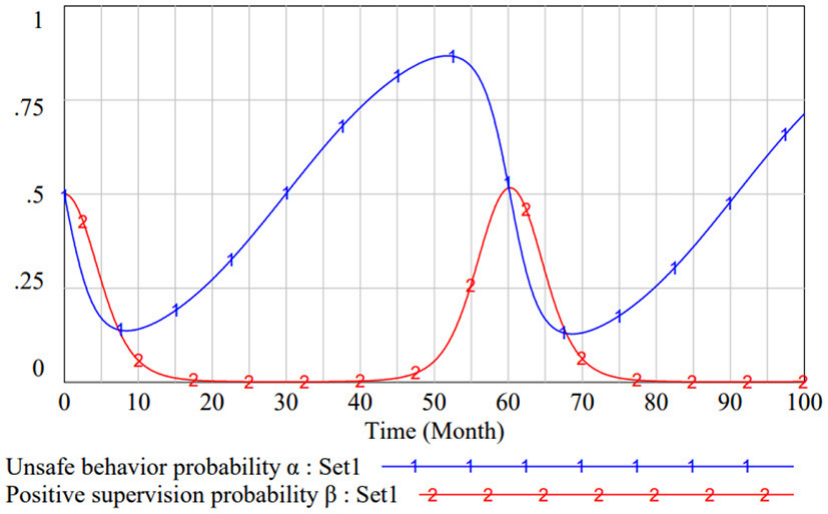
Evolutionary result of set 1.

According to the analysis above, the data of Set 1 is consistent with the Case 1 and the system has no stabilization point. The evolution result of Set 1 is shown in Figure 4, where the CWUB system fails to reach equilibrium over a period of 100 months. The two groups influence each other, and both strategies show fluctuate from one to the other. There is no evolutionary stable strategy in this game system, so the game process is difficult to control.

**Figure 5:**
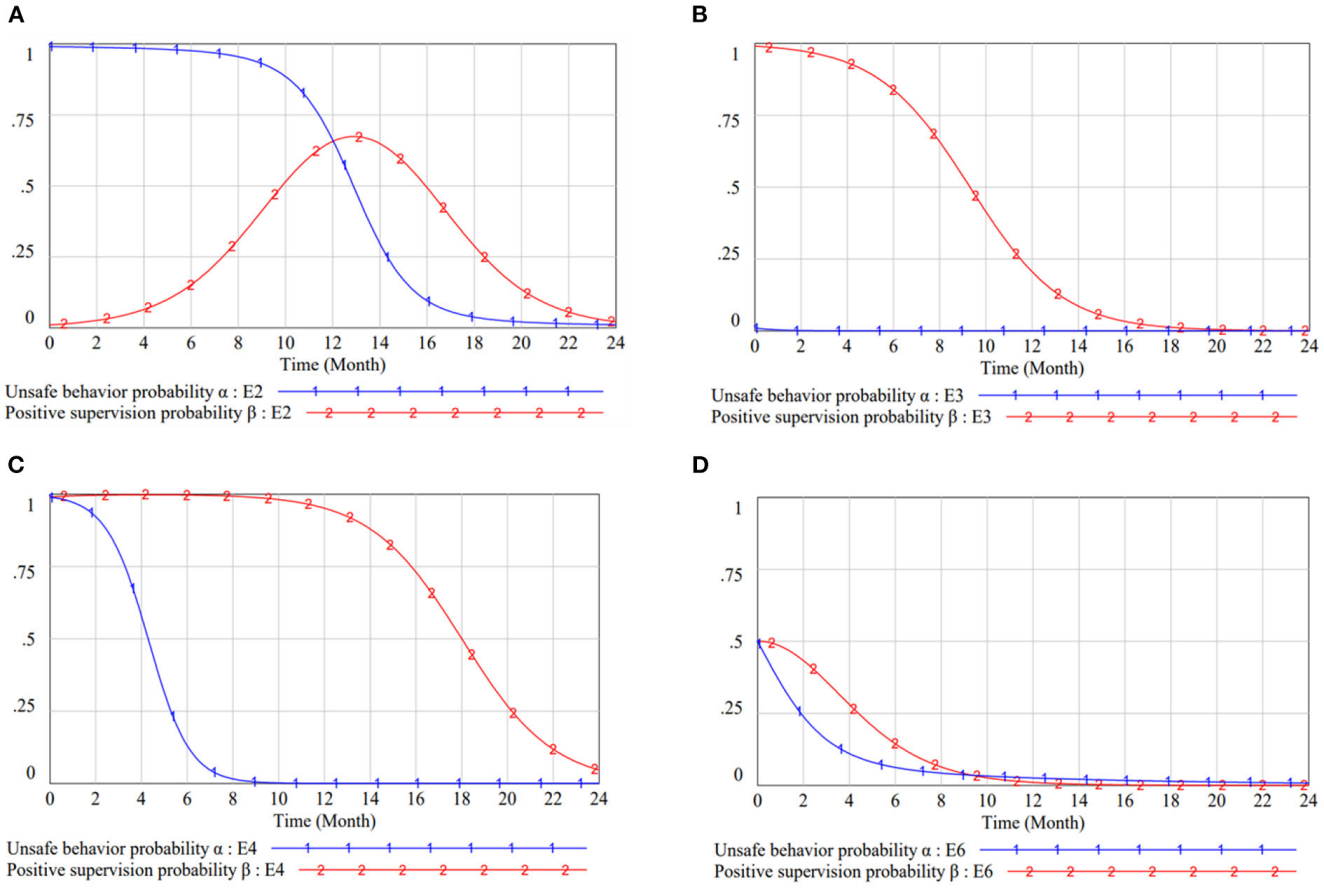
Evolutionary path of different initial strategies. **(A)** Evolutionary path of initial strategy (1, 0). **(B)** Evolutionary path of initial strategy (0, 1). **(C)** Evolutionary path of initial strategy (1, 1). **(D)** Evolutionary path of initial strategy (0.5, 0.5).

Therefore, this chapter takes the data of Set 2 and analyses the game system as the process of reaching equilibrium. With the other variables fixed, the value of the initial strategy is changed to test the evolutionary trend. In the light of Set I and Case 3, the initial strategy is simulated as three pure strategy points E2 (1, 0), E3 (0, 1), E4 (1, 1) and a mixed strategy point E6 (0.5, 0.5). In the actual software operation, the initial value setting of 0 or 1 result in no change in the curve. Based on this, we make a fine adjustment, replacing it with 0.01 and 0.99. Set the model evolution time to 24 months and TIME STEP to 0.0625. The evolutionary trend is depicted in Figure 5.

In Figure 5, curves 1 and 2 show the dynamic evaluation of the worker and the manager in the case of different initial strategies. The initial strategy does not affect their final evolutionary results, which is the same as the results of the Case 3 and Figure 2C. However, the change pattern of the curves in each graph and the time to reach equilibrium are inconsistent. Managers do not adopt negative management strategies throughout the entire process. They are “strong management” at the beginning or in the middle of the process, and take various measures to achieve a state of maximum benefit (safe behavior, negative management). While grasping the evolutionary law, finding out the reasons affecting the fluctuation of the strategies avails the management of unsafe building behavior.

In the case of Figure 5A, both subjects undergo a longer process of strategy change. At the beginning of the evolution, as construction workers adopt unsafe behaviors, the management prefers to take active control measures in order to reduce risk losses and unfavorable social opinions. Workers gradually restrain their construction behavior under the influence of safety bonuses and unsafe fines. Through 2 years of behavioral adjustment, they can eventually comply with the norms consciously. The behaviors of both parties promote each other and influence each other. Managers, knowing that workers are gradually becoming normative, tend to tilt resources elsewhere. The rate of change in workers' strategies is maximum when the probability of active management is at its peak. This also reflects the fact that in the absence of external constraints, it is difficult for workers without safety awareness to change their initial choices.

While Figure 5B illustrates the mechanism of cooperation between well-qualified workers and managers. The worker community builds a safe construction atmosphere, reduces the pressure on the management. During the game, the management has higher returns in the weakly regulated state and therefore conducts strategy updates until it is minimized. Its strategy to gain stability six months ahead of schedule. In Figure 5C, workers change their behavior 10 months earlier than in Figure 5A since managers chose to intervene at the beginning. In this case, the general trend of managers' strategy is similar to Figure 5B, but actions such as training and monitoring of workers take some months.

In contrast, when group willingness cannot be determined, we consider the probability of subjects preferring different strategies to be 0.5 for all. Figure 5D represents the strategy change at this point. If workers around them are punished for their violations and reap bonuses for compliant construction, workers will pick up on the propagation effect and abandon risky behavior. Managers as indirect participants in construction behavior, have a certain lag in behavior change, which is in line with the actual situation. Managers adopt different incentives in a state of active management to make the safety behavior of the worker group widely spread. The collection of all safety activities constitutes a good safety atmosphere on the site. In a good safety climate, workers' safety perception is stronger. When workers' safety concepts have become ingrained that managers eventually evolve to achieve safety even in a weakly regulated state, the system of unsafe behavior is broken and a virtuous circle is formed.

### Effect of external variables on strategy stability

The relevant literature and the related analysis in Table 2 show that the exogenous variables of system dynamics are taken to be critical. They can directly influence the evolutionary direction of the strategy portfolio. Sensitivity analysis of exogenous variables allows the most influential variables to be uncovered so that targeted measures can be developed (57). Based on this, we adopt a control variable approach to examine the effects of different exogenous variables on strategy choice, and test the validity of the assignment to exogenous variables. Due to the random nature of strategy choice in the group, we use the scenario in Figure 5D as the baseline scenario. In addition, the values of the exogenous variables are set to fluctuate cumulatively at a rate of 50% up and down, to simulate the trend of strategy choices (65).

Safety subsidy and unsafe penalty are factors over which management has direct control. And the impact caused by measures such as the use of emerging technologies on the site can be represented by the risk loss. Therefore, sensitivity analysis is achieved by adjusting the variables *S*, *F* and the ratio of $L_{1\;}^{1}$to $L_{2\;}^{1}$. With the original base scenario, *S*_1_−*S*_4_ are safety subsidy change scenarios, *F*_1_−*F*_4_ are unsafe penalty change scenarios, and *L*_1_−*L*_3_ are risk loss change scenarios. The specific settings are shown in Table 6.

**Table 6:** Simulation scenario setting.

**Variable**		**Variable values**		
	**Scenario**	**Safety subsidyS**	**Unsafe penalty F**	**Risk loss $L_{1}^{1}$**
Base scenario (*E*_6_)		1.2	1	0.8
Safety subsidy changes	*S*_1_	0.3	1	0.8
	*S*_2_	0.6	1	0.8
	*S*_3_	1.8	1	0.8
	*S*_4_	2.4	1	0.8
Unsafe penalty changes	*F*_1_	1.2	0.25	0.8
	*F*_2_	1.2	0.50	0.8
	*F*_3_	1.2	1.50	0.8
	*F*_4_	1.2	2.00	0.8
Risk loss changes	*L*_1_	1.2	1	0.5
	*L*_2_	1.2	1	1.2
	*L*_3_	1.2	1	1.5

For the convenience of arithmetic, the value of $L_{2\;}^{1}$is fixed and the value of $L_{1}^{1}\;$is varied to change the ratio of them.

#### Impact of safety subsidy S changes on subject strategies

**Figure 6:**
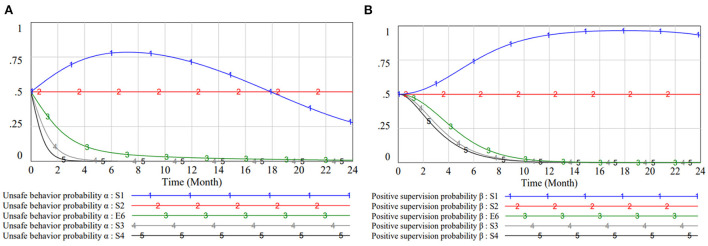
Impact of *S* on subject strategies. **(A)** Impact of *S* on workers strategy selection in different scenarios. **(B)** Impact of *S* on managers strategy selection in different scenarios.

Figure 6A reveals the probability of workers choosing unsafe behaviors in different *S* values. From the results of numerical simulations, it is clear that safety subsidies have a significant positive incentive effect on workers' behavioral norms. By increasing the subsidy at the base value, the willingness of workers to behave safely raises significantly. When S = 2.4, it takes only 4 months for workers to fully choose the “safe behavior” strategy. Once below the standard value, the curve fluctuates considerably. When S = 0.6, F(α) = 0 and F(β) = 0 is constant and the worker's strategy remains the same as the initial value. The incentive effect is limited when the bonus is too low, which makes the worker's strategy swing. Thus, the game system falls into an infinite loop and cannot reach stability.

Conversely, the safety bonus is an expense for managers. But it affects managers in a similar trend to workers, as shown in Figure 6B. This also means that the mutual influence of the game subjects is greater than the influence of the safety subsidy on the manager. But the greater the manager's incentive, the less significant the gain in strategy stability. When the payoffs are not equal to the rewards, managers also reconsider their management style. When S = 0.3, $L_{1}^{1}+S<C_{1}+L_{2}^{1}$ and *P* < *C*_2_ < *F* + *P* hold. At this time, for the sake of the reputation of the project and the atmosphere of the site, managers use the active management mode to discipline the workers in a short period of time.

#### Impact of unsafe penalty F changes on subject strategies

**Figure 7:**
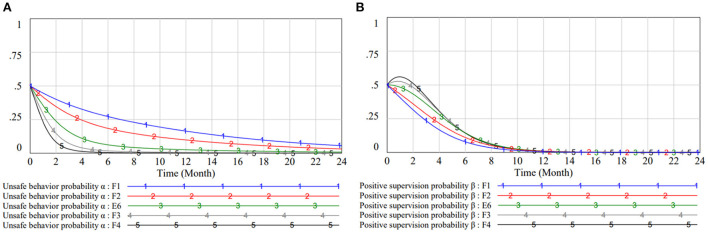
Impact of *F* on subject strategies. **(A)** Impact of *F* on workers strategy selection in different scenarios. **(B)** Impact of *F* on managers strategy selection in different scenarios.

The effect of the unsafe penalty on the worker's strategy is demonstrated in Figure 7A. The smaller the penalty, the flatter the worker's strategy curve. When the penalty takes the maximum value, workers also take only 4 months to reach strategy equilibrium. Figures 6A, 7A illustrate that in the context of this paper, the effects of both negative and positive incentives on the worker group are prominent, motivating them to switch to safe behaviors. The rational use of both incentives is the result of managerial wisdom and gaming.

Penalty is an important income for managers and a major constraint for managers on workers. As Figure 7B shows, higher fines motivate managers to actively manage in the early years. But this gain is not continuous. When fines exceed the limit of workers' affordability and make them all choose to work safely, managers need to select weak regulatory strategies to balance expenses. And fines are more sensitive to managers than workers. This is because managers are integrative in nature and workers' behavior changes more quickly. The final evolutionary results also illustrate that the variable taking is well-resistant to perturbation.

#### Impact of risk loss changes on subject strategies

**Figure 8:**
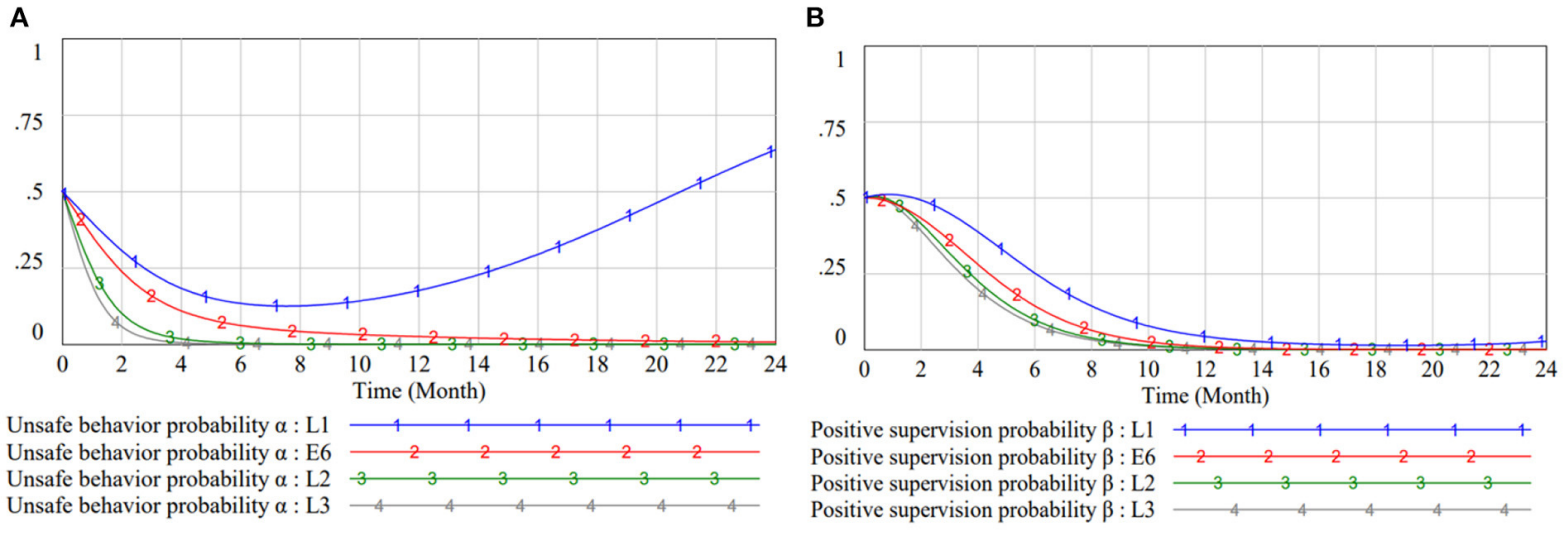
Impact of risk loss on subject strategies. **(A)** Impact of risk loss on workers strategy selection in different scenarios. **(B)** Impact of risk loss on managers strategy selection in different scenarios.

Risk loss as an indirect effect, can reflect the construction level of the project. When the risk loss ratio of unsafe behavior to safe behavior is in a high category, it indicates that safe behavior has a good risk mitigation effect. The risk-loss ratio for curve 1 of Figure 8A is 1.2:1, and the contribution of safe behavior is limited. Workers are unable to reach strategic equilibrium within 2 years and there is also a tendency to favor unsafe behavior in the long run. The threshold of the strategy turn in the chart lies roughly between 1.5 and 2. After updating the equipment and technology, introducing digital management, workers may suffer smaller values of risk loss in the state of safe behavior. In the graphs of Figure 8, the strategies of both subjects quickly evolve to the state (0, 0) as the risk-loss ratio approaches 4. This state has an ideal safety situation and the reputation loss under negative management can be reduced.

## Discussion

The CWUB system mainly involves two important stakeholders, construction workers and managers. According to the relevant theory, the system reaches the final equilibrium state under the joint action of internal and external factors. Each factor has a different effect on the payment matrix of different participants. Thus, a change in a particular influencing factor or subject's strategy can switch the direction of the system's evolution or reach a new equilibrium state. Numerical simulation reproduces the evolutionary game process between two parties, studying the paths of various factors acting on the system evolution. The results of this paper show that the system of CWUB has two direct influences, the safety subsidy and the unsafe penalty, as well as an important indirect effect, the risk loss.

In this study, the evolutionary system is constructed and numerically simulated to obtain four stability analysis scenarios, which lead to the evolution of three states. Case 2 represents the stage where the construction environment is poor and the organizational safety climate is not strong. Both workers and managers need pay more costs to contribute to the safety behavior of the group. At this time, the level of management is also insufficient, and performance evaluation and incentive mechanisms are not well-developed. Case 1 represents a transitional stage. Safety management capabilities have been improved, while the overall quality of workers has not advanced much. Management's regulatory measures make some difference, but have left the game in a state of cyclical fluctuation. While Case 3 on behalf of a stable and great organizational safety climate. Managers regulate the ratio of rewards and penalties, and workers achieve quality requirements based on cost control. CWUB emerges after the interaction of multiple factors in a complex environment. In response to the findings of the article, this section explores how to improve safety on construction sites from three perspectives: workers, managers, and the organizational.

(1) The simulation results show that reducing the initial unsafe probability of workers speeds up the evolution of their safe behavior. This also reflects the fact that improving the relevant attributes of workers can effectively curb the occurrence of unsafe behaviors. For the increasingly mechanized and intelligent construction sites, it becomes inevitable to improve the professional quality of workers. Through standardized vocational skills training, popularizing environmental awareness and safety awareness, as well as training well-performing workers to become high-end technicians, workers can be provided with endogenous motivation to have safety concepts at the early stage of construction and reduce risk losses.
(2) Managers can provide two types of behavioral incentives for workers, positive for rewards and negative for punishment. In order to keep track of the behavioral dynamics of workers, management needs to have a proven method of monitoring safety performance. Helmet removal, throwing objects from height, and equipment violations are unsafe behaviors that are not easily noticed and harmful. Since workers reach a balance of “safe behavior” very quickly when the safety allowance is increased, managers can build on this to expand the impact of it. In psychological account theory (66), when the efficiency reference point is low, workers perceive the efficiency difference strongly and thus change their strategies for high safety rewards. Restructuring workers' earnings and increasing penalties are both measures to lower the efficiency reference point.
(3) The virtuous cycle of organizational safety atmosphere and workers' safety behavior is conducive to the sustainable development of the site. Under the vigorous promotion of informatization in the construction industry, the establishment of a timely and effective information platform can enhance the overall perception of the project safety atmosphere. More importantly, the platform empowers safety managers. The safety officer at the project site no longer relies entirely on the supervision, and the safety information platform can be integrated along with the development of BIM technology. In addition, construction actors need attach importance to the feedback of information. With the problem-oriented approach, a safety supervision and mutual evaluation mechanism can be established among the teams to form a benign safety competition and strengthen the safety atmosphere of the organization.

## Conclusions

CWUB is an important reason for the frequent occurrence of safety accidents. A systematic sorting out of CWUB can help explore a reasonable governance path. For the complex characteristics of construction behavior, we got the relevant covariates of behavior evolution. And under the premise of limited rationality, we explored their influence on the behavior between workers and managers through SD evolutionary game model. Based on this study, we draw the following four conclusions, which are meaningful for promoting the construction site safety.

(1) In the evolutionary game process of CWUB, there are two equilibrium states and one unstable state. When the bonus given by the manager cannot fill the cost of safe construction and the penalty is relatively large, the evolutionary game system falls into a chaotic state. On the contrary, if the amount of safety bonuses is substantial, positive incentives guide the strategies of both parties toward safe behavior and weak management. When safe behavior and active management cost more, it leads to a poor safety climate. Workers choose to violate the rules and managers not on board.
(2) Different initial strategies make the time to reach equilibrium for the strategies of the two game subjects inconsistent. Managers stand in an integrated perspective and are the main regulators of the safety climate, with a lag in their strategic shifts. A well-trained, safety-conscious workforce can help managers save at least half the time in decision making, thus optimizing the overall resource allocation for the project. Group behavior has a network propagation effect, and the virtuous circle formed by a good safety atmosphere and continuous safety behavior is conducive to the sustainable development of construction.
(3) For the strategy choice of workers, positive and negative incentive policies of managers play an important role (67). A high safety allowance is a driving force for workers' choice of safe behavior, while a high unsafe penalty is a binding force for workers' choice of safe behavior. The construction techniques adopted by workers entail a corresponding risk loss, which is an indirect influence variable. Reducing risk loss when working safely is another powerful way to motivate indecisive groups of workers to switch to safe behaviors.
(4) In different contexts, managers' strategic choices are relevant to workers. Important factors such as safety bonus, unsafe penalty and risk loss are mainly reflected to managers by influencing workers' behavior. Among them, safety bonus and risk loss are positively related to willingness to actively manage, and there is no significant difference in sensitivity. And fines, as a benefit to managers, strengthen the probability of active management in the short run. However, workers' choice has the highest degree of influence on managers' choice. In the set context, managers eventually choose a negative management strategy.

This paper analyses the equilibrium strategy of the CWUB system composed of both construction workers and managers. The results of the study provide some valuable references for enhancing the safety climate at construction sites. However, the limited conditions leave some areas for improvement. Different industrial policies have led to different key stakeholders in CWUB. The power interest matrix can be relied upon for the expansion and identification of game subjects. Secondly, methods such as table functions can compensate for the uncertainty caused by time. In the future, quantitative analysis methods such as deep learning and intelligent detection can be combined to increase the credibility of the research.

## Data availability statement

The original contributions presented in the study are included in the article/supplementary material, further inquiries can be directed to the corresponding author.

## Author contributions

JH and YW had the idea for the article. JH conducted assumptions and simulation design. YW built the SD model. YH, YY, and GG performed the literature search and data collection. JH, YW, YH, and HC drafted the manuscript. All authors contributed to the article and approved the submitted version.

## Funding

This research was funded by the National Natural Science Foundation of China (Grant No. 71942006) and the Sichuan Province Science and Technology Project (Grant No. 2020JDR0396).

## Conflict of interest

The authors declare that the research was conducted in the absence of any commercial or financial relationships that could be construed as a potential conflict of interest.

## Publisher's note

All claims expressed in this article are solely those of the authors and do not necessarily represent those of their affiliated organizations, or those of the publisher, the editors and the reviewers. Any product that may be evaluated in this article, or claim that may be made by its manufacturer, is not guaranteed or endorsed by the publisher.
